# Capturing biomarkers associated with Alzheimer disease subtypes using data distribution characteristics

**DOI:** 10.3389/fncom.2024.1388504

**Published:** 2024-09-03

**Authors:** Kenneth Smith, Sharlee Climer

**Affiliations:** Department of Computer Science, University of Missouri – St. Louis, St. Louis, MO, United States

**Keywords:** Alzheimer disease, precision medicine, subtypes, biomarkers, association studies, fold change, AUC, bimodality

## Abstract

Late-onset Alzheimer disease (AD) is a highly complex disease with multiple subtypes, as demonstrated by its disparate risk factors, pathological manifestations, and clinical traits. Discovery of biomarkers to diagnose specific AD subtypes is a key step towards understanding biological mechanisms underlying this enigmatic disease, generating candidate drug targets, and selecting participants for drug trials. Popular statistical methods for evaluating candidate biomarkers, fold change (FC) and area under the receiver operating characteristic curve (AUC), were designed for homogeneous data and we demonstrate the inherent weaknesses of these approaches when used to evaluate subtypes representing less than half of the diseased cases. We introduce a unique evaluation metric that is based on the distribution of the values, rather than the magnitude of the values, to identify analytes that are associated with a subset of the diseased cases, thereby revealing potential biomarkers for subtypes. Our approach, Bimodality Coefficient Difference (BCD), computes the difference between the degrees of bimodality for the cases and controls. We demonstrate the effectiveness of our approach with large-scale synthetic data trials containing nearly perfect subtypes. In order to reveal novel AD biomarkers for heterogeneous subtypes, we applied BCD to gene expression data for 8,650 genes for 176 AD cases and 187 controls. Our results confirm the utility of BCD for identifying subtypes of heterogeneous diseases.

## 1 Introduction

Advances in precision medicine (PM) for cancer patients are extending the healthspan for countless lives by tailoring treatments to heterogeneous cancer subtypes. PM utilizes specific biomarker information to diagnose each specific subtype of the disease and enable customized treatments, prognoses, and monitoring. Candidate biomarkers may include genetics, demographics, lifestyle, and/or physiological observations such as imaging or omics data (e.g., levels of gene expression, proteins, lipids, or metabolites). An additional benefit of PM is that it facilitates understanding of underlying biological mechanisms by teasing apart biomarkers into subtype groups. Knowledge of distinct biomarkers associated with each subtype empowers drug discovery as well as selections of participants for drug trials.

Heterogeneous subtypes of late-onset Alzheimer disease (AD) are exhibited by the disparate genetic and environmental risk factors and clinical outcomes observed for this enigmatic disease. Efforts are underway to enable PM for AD, including the Accelerating Medicines Partnership® for AD 2.0 (Accelerating Medicines Partnership, 2022), which began in 2021, and Alzheimer Precision Medicine Initiative (Hampel et al., 2019), which began in 2016.

Imaging methods and CSF total tau (tTau) have been used to discriminate typical and atypical AD subtypes associated with brain regions. While positive amyloid PET indicates AD status in general, fluorodeoxyglucose PET (FDG-PET), and tau ligand binding suggest five subtypes: typical amnestic syndrome, logopenic variant of primary progressive aphasia, posterior cortical atrophy, corticobasal syndrome, and frontal AD (Dubois et al., 2023). Furthermore, Pillia et al. observed an association between the upregulation of tTau in CSF and the atypical logopenic variant subtype (Pillai et al., 2019).

Ferreira et al. conducted an extensive meta-analysis of neuropathology and neuroimaging studies and propose AD subtypes based on two dimensions: typicality and severity (Ferreira et al., 2020). The four subtypes are typical AD, limbic-predominant, hippocampal-sparing, and minimal atrophy. They present covariates that are associated with the two subtypes at the extremes of the typicality dimension, limbic-predominant and hippocampal-sparing.

Recently, deep learning methods incorporating multiple data types, such as imaging, omics data, and clinical assessments, have introduced multimodal models. For example, Reyes et al. proposed a tri-modal co-attention transformer, referred to as Tri-COAT, to classify AD cases into three progression-specific subtypes (Machado Reyes et al., 2024). They integrated imaging data, genetics, and clinical records by using transformer modules to encode each type separately, then merging the three into a co-attention model to learn feature weights and relationships across the three data types.

Nevertheless, PM progress has been limited for AD as well as a host of additional neurological diseases. Unlike cancer, which provides a written history of mutations, diseased and healthy cells for comparisons from a given patient, and excellent animal models for experiments, pathological clues for AD lie buried deep in the human brain with only traces of evidence that leak into peripheral systems.

The realization of successful PM can only be attained by identifying disease subtypes and developing practical methods to diagnose and treat each subtype. A common first step is to use statistical methods to test associations of candidate biomarkers with the disease. Different statistics are used for categorical, ordinal, and numerical data types. Herein we focus on numerical data types, which includes omics data (e.g., gene expression and protein levels), measurements from imaging data (such as PET amyloid load), and other observations that are quantified as numerical values. Popular statistics for this domain include fold change (FC) of levels of candidate biomarkers between diseased cases and normal controls and area under the receiver operating characteristic curve (AUC; Xia et al., 2013).

The nascent PM for AD research field faces challenges due to multiple issues, including the need for large sample sizes to elicit power to sift out a subtype that may only represent a small fraction of the diseased cases. An overlooked, but major, challenge is that traditional statistical methods that are successful for global biomarkers can be inappropriate for subset biomarker identification. Stated bluntly, traditional methods need to be scrutinized for use in this distinct domain.

In order to assess current statistical metrics for advancing PM for AD, we examine the use of FC and AUC when subtype groups exist. FC is a traditional approach for identifying analytes that are differentially expressed across diseased cases and normal controls. It is equal to the quotient of the analyte expression levels between the two groups: (level in diseased cases)/(level in normal controls). If the quotient is above or below a given cutoff, the analyte is considered differentially expressed. A single value representing the expression level of the analyte is required for each group; usually the median or mean. Typically, a cutoff of >2 is used to indicate significant up-regulation in the diseased cases group and a cutoff of <0.5 for down-regulation. In order to more easily interpret across both up-and down-regulated analytes, the log2FC is often employed, where log2FC = abs{log_2_[(level in diseased cases)/(level in normal controls)]}, providing a significance threshold of log2FC > 1 for both up-and down-regulation (Pacholewska, 2017). Some weaknesses of this metric have been previously noted. FC calculations are unstable when the expression levels are near the noise level of the measurement system. This can lead to false positives at low intensity levels. At the other end of the spectrum, FC is also biased against samples that have high expression levels, but small differences between two groups (Mariani et al., 2003). Mariani et al. reported that high FC cutoffs are needed for low intensity genes and lower cutoffs are needed for high intensity genes. They introduced a variable FC cutoff-based approach that uses LOESS to estimate a variance based on expression intensity, thereby alleviating the bias at both high and low intensity levels (Mariani et al., 2003). Despite these improvements to the FC calculation, there is a fundamental problem with this metric: Use of the mean or median in the presence of heterogeneity leads to the omission of subgroup signals, as demonstrated in this manuscript.

Standard 2 × 2 contingency tables are commonly used to assess predictive accuracy of biomarkers using various statistics, such as sensitivity/specificity, precision/recall, Fisher’s Exact Test (Fisher, 1935), and Youden’s J index (Youden, 1950). Note that Youden’s J definition can be rearranged to produce a simple interpretation: J = TPR − FPR, where TPR is the true positive rate and FPR is the false positive rate. A key benefit of utilizing Youden’s J is that subgroups can be captured, rather than being lost in a summary statistic, as is done with FC. However, without other information, subgroups may be overlooked due to the existence of moderate case/control biomarkers with the same J value, just higher TPR and FPR, e.g., J = 0.20 − 0.01 vs. J = 0.70 − 0.51. Importantly, in order to classify real values as true or false positives, a threshold must be designated, and Youden’s J value is highly dependent upon the given threshold.

More generally, when testing numerical values, 2 × 2 contingency tables require the selection of a threshold to separate diagnostic classifications. A key strength of AUC is that it has no reliance upon a specified threshold. This metric originated as a tool for radar receivers, spread throughout engineering and medical domains, and has become a prevalent tool for evaluating the diagnostic ability of biomarkers (Xia et al., 2013; Zweig and Campbell, 1993; Sø, 2009). AUC simultaneously accounts for sensitivity and specificity across all threshold values as a plot of the TPR vs. FPR is constructed and the area under the curve is returned as the AUC value (Pepe, 2000). The plot for a random classifier would tend toward a diagonal line from (0,0) to (1,1) with an AUC value of 0.5. A ‘perfect’ predictor would have FPR = 0 and TPR = 1 for all thresholds of the biomarker and a corresponding AUC value of 1. An example of this rare event was reported by Karikari et al. for discriminating Alzheimer disease from healthy young adults using plasma tau phosphorylated at threonine 181 (pTau-181) (Karikari et al., 2020).

There is not a consensus for a significance cutoff for AUC values. Previous publications have suggested an AUC between 0.7 and 0.8 as acceptable and greater than 0.8 as excellent (Cucchiara, 2013; Mandrekar, 2010), while the National Center on Response to Intervention’s Technical Standard sets AUC values between 0.75 and 0.85 as ‘partially convincing’ and below 0.75 as ‘unconvincing’ (Bowers and Zhou, 2019). On the other hand, it has been recommended that no set value should be utilized; rather AUC values should be used to compare predictors within a single domain rather than enforcing a strict cutoff value (Zweig and Campbell, 1993; Hanley and McNeil, 1982; Swets, 1988; Bowers et al., 2013).

In addition to evaluating biomarkers across all threshold values, AUC has several other beneficial properties. It is a simple and intuitive measure, and the corresponding ROC plot provides additional information beyond the scalar value. Also, there are no parameters to be tuned, yielding robust reproducibility.

There are also some well-known issues with AUC. First, small sample size can yield poor performance (Hanczar et al., 2010; Dudbridge, 2013). Second, AUC includes the areas under the ROC curve that represent threshold values that are not utilized in practical applications (Lobo et al., 2008). A related issue is when the ROC curves of two different biomarkers cross, the relative AUC values may be misleading (Hand, 2009).

In general, the points in the ROC curve arise *solely* from differences in TPR and FPR and are not scaled across threshold values, resulting with the possibility of a small span of threshold values being stretched across broad regions of the area under the curve. Consequently, a small difference in the level of the analyte would correspond to large differences in specificity and sensitivity. In clinical practice, target thresholds or threshold ranges are used to flag individuals at risk. AUC values are generally computed over clean data that have been acquired and processed using highly uniform methods, but this uniformity deteriorates when moving from bench to bedside. In general, examination of AUC values and plots may not directly provide insights for selecting a suitable diagnostic threshold that is robust across measurement error. The metric introduced in this manuscript addresses this issue.

The AUC metric is entirely dependent upon, and equally weighted on, the TPR and FPR. When testing across a heterogeneous group, an accurate TPR for a perfect biomarker has an upper limit equal to the proportion of the subtype. Due to its dependence upon TPR, we hypothesize that screening based on AUC may discard valuable subtype biomarkers, regardless of sample size. Using simulated tests mimicking nearly ‘ideal’ biomarkers for subsets of disease cases, we demonstrate the failure of AUC to capture their significance.

The need for a robust evaluation metric in the heterogeneous AD domain inspired us to design a tool that is based upon the *distribution* of values, rather than traditional statistical measurements. Consider a biomarker that is a strong indicator of a subset of diseased cases, referred to as ‘associated cases’. We assume here that the cases that are not part of this subtype exhibit biomarker levels that are similar to the normal controls. Consequently, the distribution of biomarker levels for the cases tends to skew the distribution or exhibit a bimodal profile, where one of the modes lines up with the controls’ distribution.

It should be noted that normal controls might show a bimodal distribution also. For example, blood sugar levels are high following a meal and low just before a meal, so controls sampled at varying times of day would be prone to exhibit a bimodal curve for this analyte.

Aiming to identify aberrant bimodal distributions, we propose a metric which calculates the *difference* between the bimodalities of the diseased cases and normal controls. The first task is to select a method to measure the degree of bimodality for an array of data values. A number of formulae for distinguishing between unimodality and bimodality have been previously proposed and evaluated (Freeman and Dale, 2013). Hartigan’s Dip Statistic (HDS) (Hartigan and Hartigan, 1985) and the Bimodality Coefficient (BC) (SAS Institute Inc, 1990) have both been shown to have good accuracy to detect bimodality (Freeman and Dale, 2013). Note that high skewness in a unimodal distribution tends to increase BC values and can lead to false-positive bimodal predictions (Pfister et al., 2013). We selected BC as we are interested in identifying either bimodality or skewness that is significantly different between cases and controls.

We introduce Bimodality Coefficient Difference (BCD) as the difference in the BC values for the diseased cases and normal controls. BCD can theoretically range from zero to one, but we observe in our trials that relatively low values indicate significance. Using a series of simulation trials, we demonstrate the effectiveness of this metric over FC and AUC for identifying analytes with clear subtype populations that comprise less than half of the simulated cases. We then leverage this method in an analysis of AD gene expression data and reveal known and novel genes exhibiting bimodal distributions for the AD cases. Notably, more than 95% of the genes discovered by BCD were missed by both FC and AUC. The python software package for computing BCD is freely available at: https://github.com/ClimerLab/bcd.

## 2 Methods

### 2.1 BCD

The Bimodality Coefficient, BC, was introduced by SAS in 1990 and is based on three parameters of the array of values: cardinality (*n*), skewness (*s*), and kurtosis (*k*). The value is computed as follows:


$$BC=\frac{s^{2}+1}{k+3\times \frac{{\left(n-1\right)}^{2}}{\left(n-2\right)\left(n-3\right)}}$$

BC values range from zero to one and a uniform distribution has a value of 5/9 ≈ 0.555. Higher values indicate greater bimodality.

We propose the following measure, bimodality coefficient difference (BCD), for identifying biomarkers representing subtypes in heterogeneous populations:


$$BCD=|BC_{\mathrm{cases}}-BC_{\mathrm{controls}}|$$

The absolute value is applied as a protective factor may result with the controls having a higher BC value than the cases.

### 2.2 Simulated data trials

**Figure 1:**
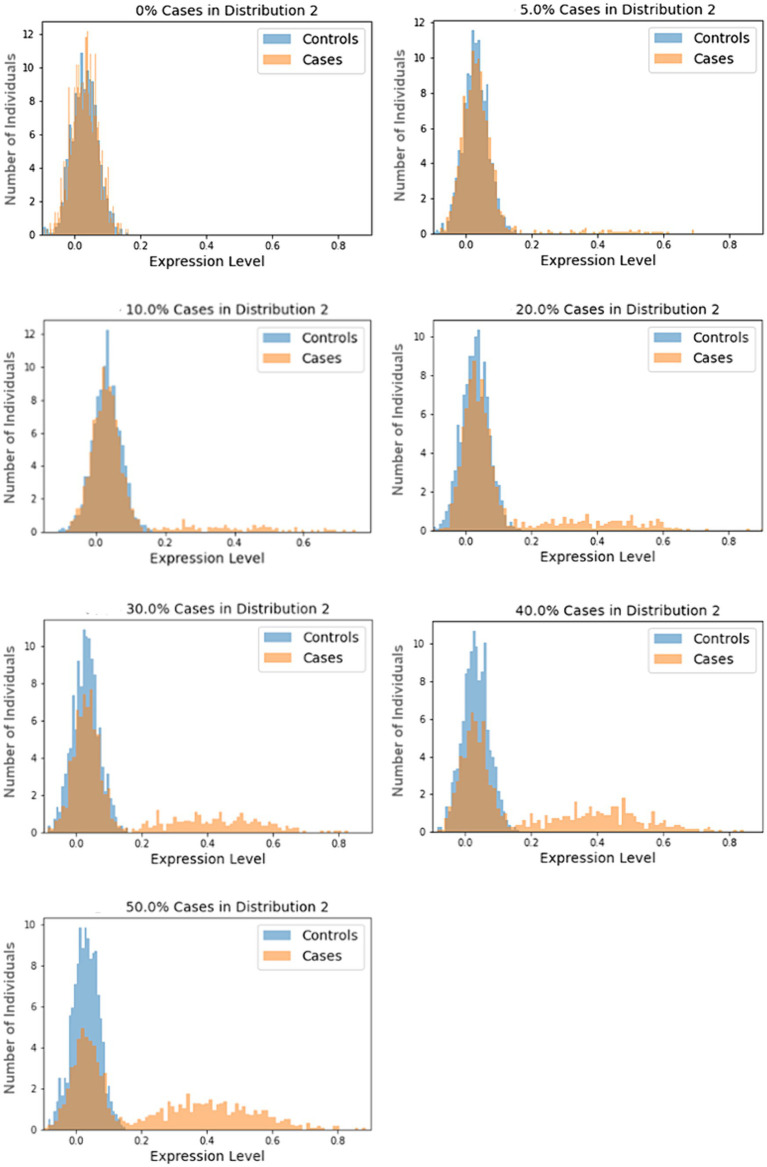
Sample histograms for the simulation trials. Shown are random histograms drawn from the 1,000 trials for each of the seven subset size scenarios.

In our simulations, samples are drawn from one of two normal distributions, N_1_ and N_2_, with the following means and standard deviations: N_1_ ~ (0.03, 0.04) and N_2_ ~ (0.40, 0.16). These means and standard deviations were derived from analysis of highly differentially-expressed proteins from our COVID-19 study, as described in the Supplementary material. The size of the subtype, as a percentage of the cases, are varied over seven scenarios from 0 to 50%. In each scenario, the cases in the subtype group were sampled from N_2_ and the remaining cases, along with all controls, are sampled from N_1_. A total of 1,000 cases and 1,000 controls are simulated in each trial. Each scenario was tested using 1,000 trials. Histograms for randomly selected trials are shown in Figure 1.

### 2.3 Biological data trials

We utilized publicly-available gene expression data from human cortex tissue generated using Sentrix HumanRef-8 Expression BeadChip (Webster et al., 2009). These data are available on NCBI’s Gene Expression Omnibus (GEO), Accession GSE15222. Standard protocols for cRNA hybridization and BeadStudio software, with Illumina’s custom error model, were utilized in data generation, as previously described (Webster et al., 2009). Data for 8,650 genes for 176 AD cases and 187 controls are provided and used for the current study.

Following analyses utilizing FC, AUC, and BCD, the highest 5% results were extracted for each method and used for comparisons between the methods. In order to further interrogate the results and examine data distributions, the six best genes for each method were extracted and plotted. Note that multiple testing corrections were not applied for any of the methods and the presented results need to be validated in independent data prior to further research effort.

### 2.4 Data pre-processing

The AD data were pre-processed by the Myers’ lab, as described previously (Webster et al., 2009). Outliers disproportionately affect BC values and there is no clear consensus on eliminating them prior to computing BC. Here we winsorized the outliers as follows. Given a lower quartile Q1, upper quartile Q3, and IQR, values higher than Q3 + 3*IQR were replaced with Q3 + 3*IQR and values less than Q1 - 3*IQR were replaced with Q1-3*IQR.

Because the simulated data can contain ‘negative’ expression values, a min/max normalization was applied for FC calculations and plotting to scale and shift the values to a range of [0, 1]. Also, a logistical regression model was generated in the AUC computations.

## 3 Results

### 3.1 Simulation trials

We generated large-scale simulated data for a total of 7,000 pseudo analytes over a range of subtype percentages and analyzed each using FC, AUC, and BCD. The subset size of zero provides a baseline for which no association should be observed as all the data points for cases and controls are drawn from the N_1_ distribution. The other trials test subset sizes of 5, 10, 20, 30, 40, and 50%. Results for the simulations are summarized in Table 1. As expected, *Sim_0%*, with none of the data values drawn from N_2_, yielded values near zero for log2FC and BCD, and near 0.5 for AUC.

**Table 1:** Median values for the simulation trials, with minimum and maximum values shown in brackets.

Trial	log2FC	AUC	BCD (>2.09)
*Sim_0%*	0.016 [5.47E−06, 0.080]	0.508 [0.491, 0.542]	0.016 [8.68E−06, 0.076]
*Sim_5%*	0.0276 [2.25E−06, 0.107]	0.525 [0.483, 0.563]	0.145 [0.066, 0.214]
*Sim_10%*	0.0544 [2.79E−04, 0.143]	0.548 [0.509, 0.605]	0.282 [0.214, 0.350]
*Sim_20%*	0.124 [0.037, 0.210]	0.598 [0.563, 0.630]	0.395 [0.325, 0.458]
*Sim_30%*	0.206 [0.071, 0.320]	0.646 [0.617, 0.675]	0.441 [0.372, 0.514]
*Sim_40%*	0.337 [0.134, 0.452]	0.695 [0.669, 0.723]	0.464 [0.389, 0.525]
*Sim_50%*	0.608 [0.221, 0.821]	0.743 [0.719, 0.768]	0.438 [0.383, 0.504]

The first row represents no subsets, where all of the cases and controls values are drawn from the N_1_ distribution. Subsequent rows represent trials with 5, 10, 20, 30, 40, and 50%, respectively, of the cases values drawn from the N_2_ distribution and represent the disease subtype. For each scenario, 1,000 cases and 1,000 controls values were generated for each of 1,000 trials. Note that the BCD analysis of the AD gene expression data provides a threshold of 0.209 for *p*-value ≤0.05.

**Figure 2:**
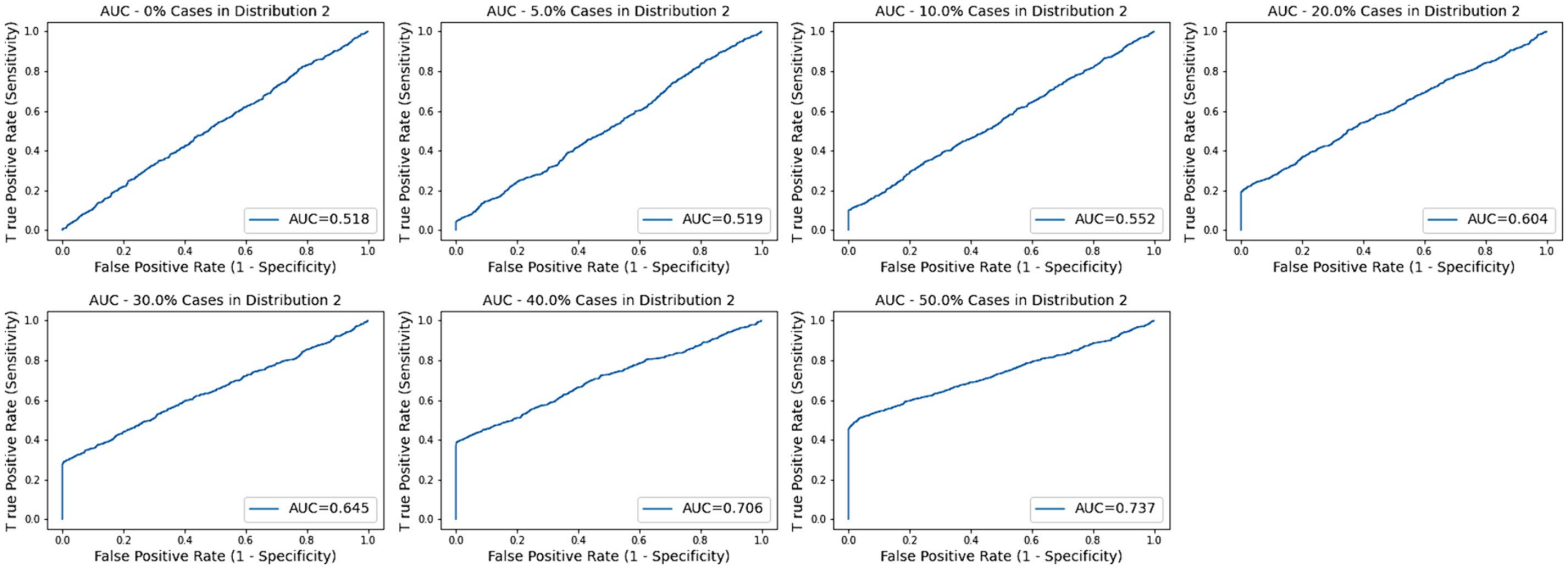
AUC plots for subtype groups of 0, 5, 10, 20, 30, 40, and 50% of the diseased cases. Random plots for each scenario are shown.

Across the remaining trials with subtypes ranging from 5 to 50%, none of the log2FC values were significant as the maximum value across all the simulations is 0.821. None of the AUC values were significant for subsets of 30% or less as the highest across those simulations was 0.675. The medians for subset size 40 and 50% were 0.695 and 0.743, respectively. As described below, the AD data provided a threshold of 0.209 for a *p*-value of 0.05 for BCD. Based on this proxy, BCD values were significant for all trials with subset sizes of 10% or more as well as a few of the 5% subset size. Sample ROC curves for each scenario are shown in Figure 2. Note the vertical rise on the left, which perfectly captures the subtype, followed by the relatively straight diagonal line across the graph.

### 3.2 Alzheimer disease data

In our first round of BCD trials, the genes with the highest values proved to be spurious. For example, gene GI_37540877-S exhibited the strongest association, with an BCD value of 0.377. This signal was erroneous, as described next.

**Figure 3:**
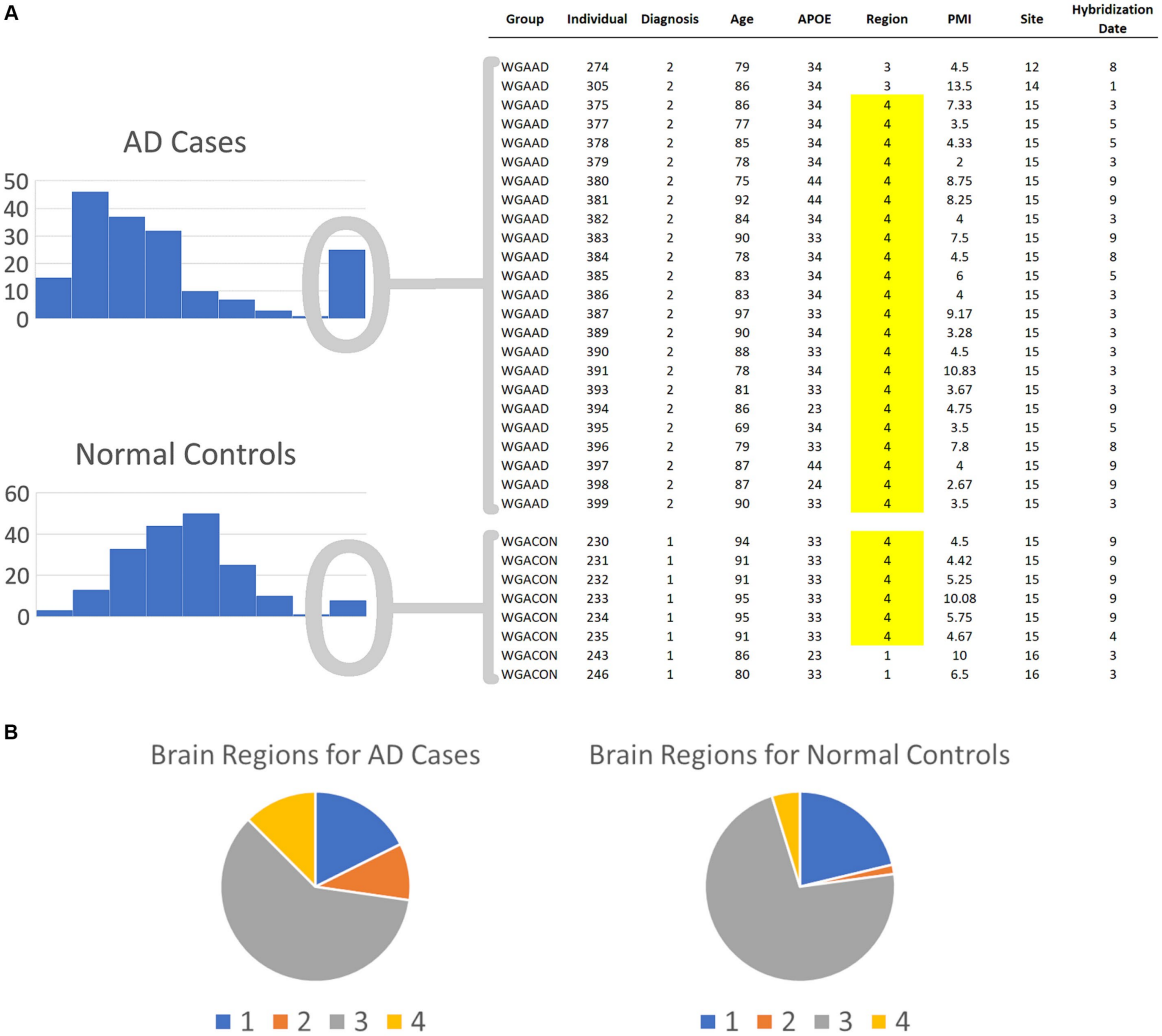
Spurious results for the first round of BCD trials on the AD biological data. **(A)** The histograms depict the numbers of AD cases and normal controls with each expression value range for the most significant BCD gene, GI_37540877-S. The covariates for the individuals in the second mode are shown on the right. Note that 91.7 and 75.0% of the cases and controls, respectively, are samples from brain region 4. **(B)** Overall, brain region 4 comprises only 12.5 and 4.8% of the cases and controls, respectively. These results suggest that BCD identified differences between AD cases and normal controls due to the differences in the expression of GI_37540877-S in the various brain regions.

Both the AD cases and normal controls have outlier values for this gene and these outliers were winsorized, as described in the Methods section, to values of 331.65. As shown in Figure 3, 24 of the AD cases and 8 of the normal controls exhibit these outlier values for this gene. We extracted the covariate data for these samples and observed all but two in each group were brain samples drawn from region 4 (Figure 3). 91.7% of the cases in the second mode were drawn from brain region 4, even though only 12.5% of the case samples overall were drawn from this region. Furthermore, only 4.8% of the control samples were drawn from region 4, yielding a strong imbalance of samples for this region. Consequently, diseased cases samples that were drawn from region 4 form distinct subsets that create second modes for genes that are differentially expressed across the brain regions. These results demonstrate the power of BCD to identify subtypes, but do not yield information of interest regarding AD. As shown in the Supplementary Table S1, brain region 2 is also unbalanced between cases and controls.

In our second round of trials, we removed samples drawn from brain regions 2 and 4, yielding 137 AD cases and 175 normal controls then analyzed the data using FC, AUC, and BCD. The genes with the highest 5% of values for each method are enumerated in Supplementary Tables S2–S4. Across the 8,650 genes, FC, AUC, and BCD values of 0.637, 0.740, and 0.209, respectively, represented the cutoffs for *p*-values ≤ 0.05.

Overall, 46.1% of the significant genes for FC and AUC were the same. In sharp contrast, only 3.7 and 4.6% of the significant BCD genes were identified by FC and AUC, respectively. Overall, 2.3% of the significant genes for each method were common across all three approaches (Supplementary Table S5).

**Figure 4:**
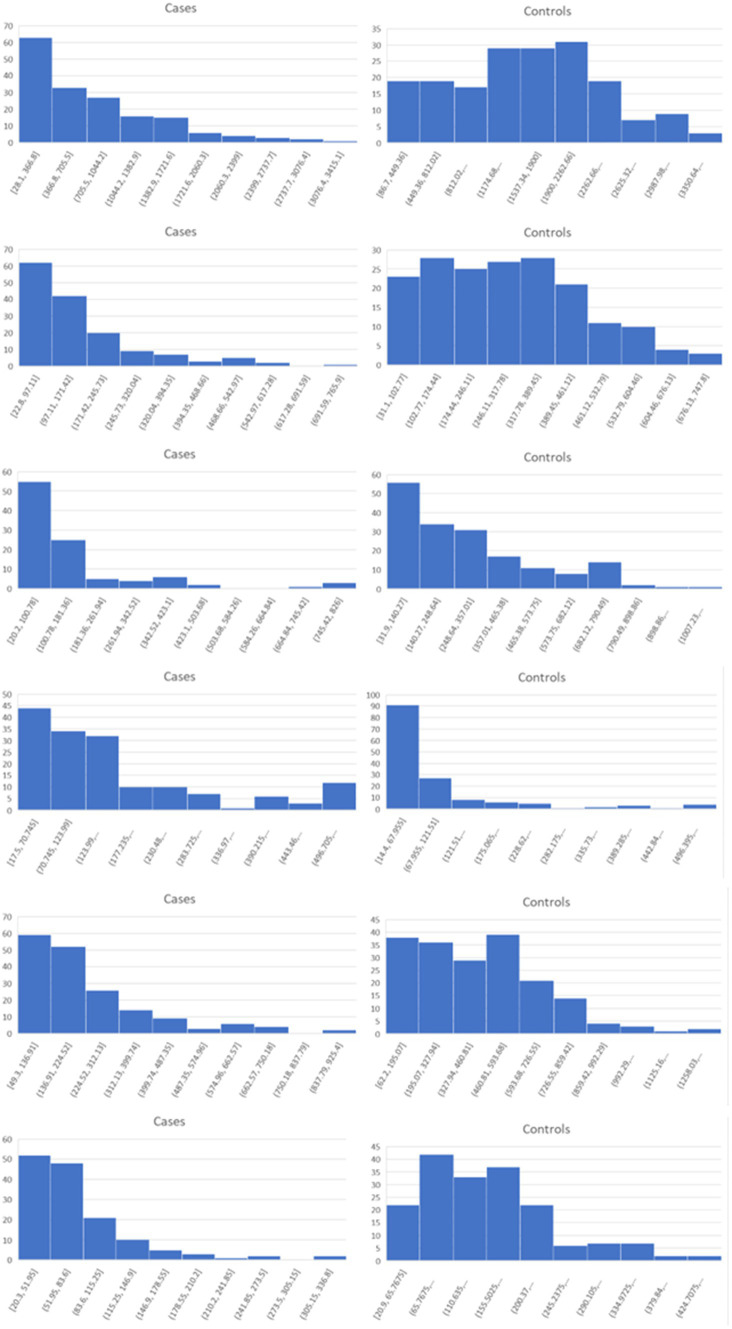
Histograms for the six top genes identified using FC. Each row corresponds to a gene in Table 2 and are given in the same order.

**Figure 5:**
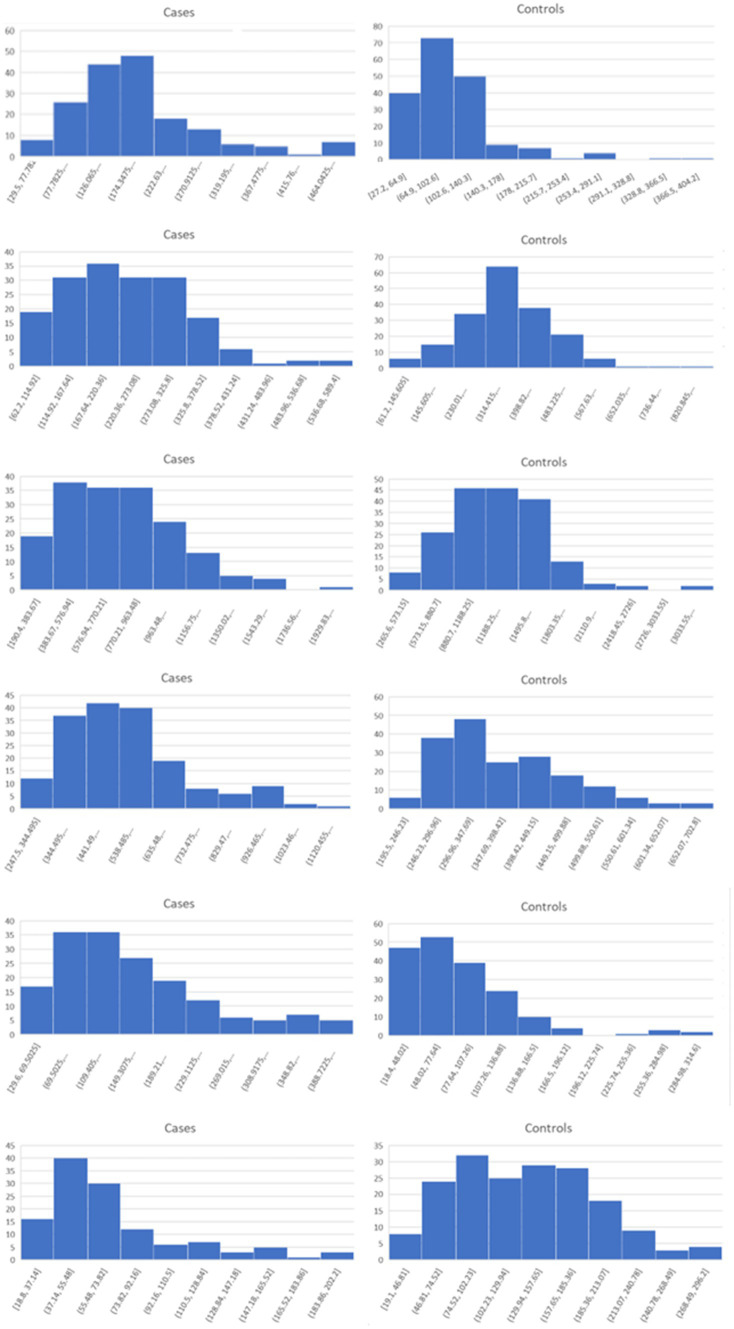
Histograms for the six top genes identified using AUC. Each row corresponds to a gene in Table 2 and are given in the same order.

**Figure 6:**
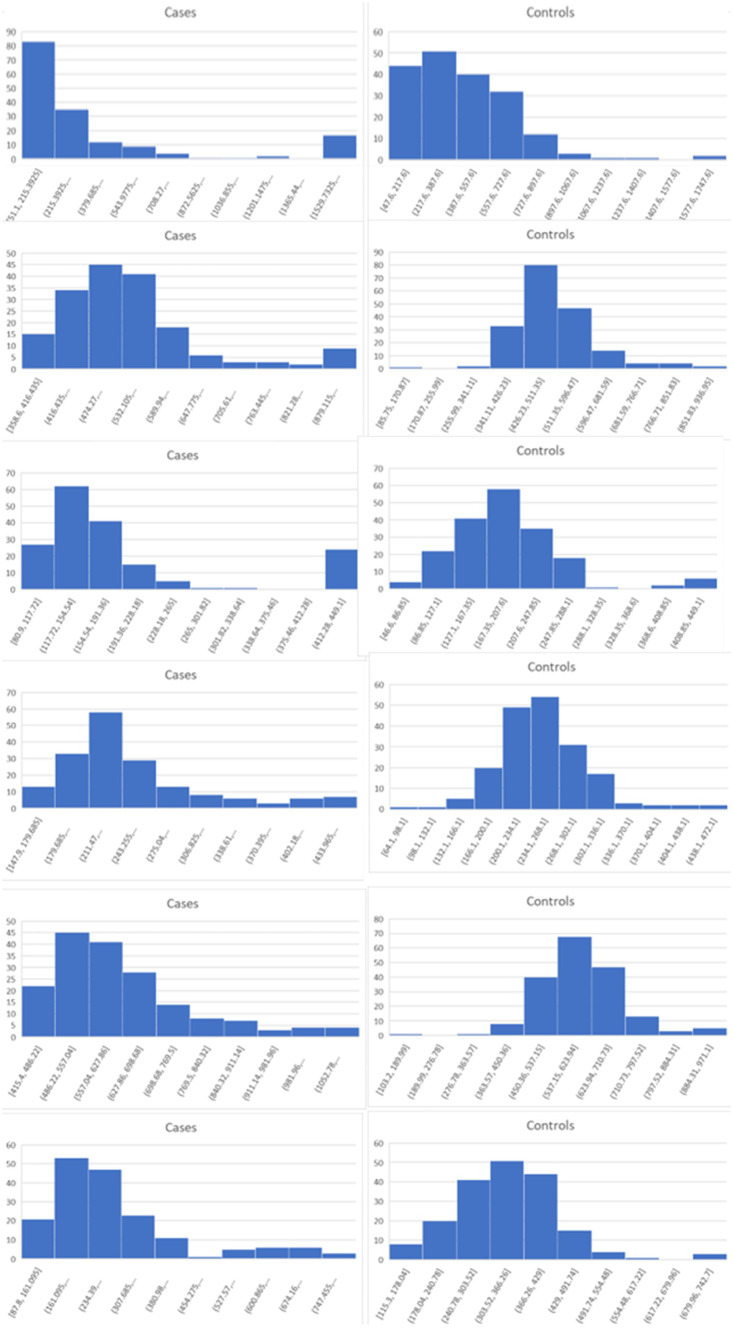
Histograms for the six top genes identified using BCD. Each row corresponds to a gene in Table 2 and are given in the same order.

Lists of the top six genes for FC, AUC, and BCD are shown in Table 2, histograms for each of these genes are shown in Figures 4–6 and Table 3 provides descriptions of the top AD genes identified by BCD. Some of the FC and AUC plots exhibit tendency towards bimodality or increased skew, but in general they represent differences in expression across the majority of the samples, demonstrating their value for identifying biomarkers associated with large proportions of the cases.

**Table 2:** Top six genes for each analysis of the AD gene expression data.

Fold Change			AUC			BCD		
GeneID	Gene	log2FC	GeneID	Gene	AUC	GeneID	Gene	BCD
GI_38201693-S	RGS4	1.448	GI_4585642-S	ZNF264	0.854	GI_4502806-S	CHGB	0.403
GI_40255112-S	MGC35285	1.428	GI_27734844-S	ZDHHC23	0.830	GI_17999536-S	PRPF8	0.378
GI_40254432-S	N/A	1.342	GI_24308166-S	DKFZp761H039	0.829	GI_37540877-S	GLCE	0.378
GI_40018630-A	N/A	1.306	GI_34577121-S	NFKB1	0.822	GI_28872783-A	CDK5RAP1	0.375
GI_29744077-S	LOC340542	1.288	GI_13376557-S	FLJ21272	0.820	GI_14589948-S	POLR2A	0.374
GI_27475984-S	NEUROD6	1.217	GI_23312375-A	PPEF1	0.815	GI_23503234-A	C1QDC1	0.372

**Table 3:** Descriptions of the top six genes identified by BCD in the AD data.

GeneID	Gene	Description	Location	Alias	NCBI Summary
GI_4502806-S	CHGB	Chromogranin B (secretogranin 1)	20p12.3	SCG1	This gene encodes a tyrosine-sulfated secretory protein abundant in peptidergic endocrine cells and neurons. This protein may serve as a precursor for regulatory peptides (provided by RefSeq, January 2009).
GI_17999536-S	PRPF8	Pre-mRNA processing factor 8	17p13.3	PRP8; RP13; HPRP8; PRPC8; SNRNP220	Pre-mRNA splicing occurs in 2 sequential transesterification steps. The protein encoded by this gene is a component of both U2-and U12-dependent spliceosomes, and found to be essential for the catalytic step II in pre-mRNA splicing process. It contains several WD repeats, which function in protein–protein interactions. This protein has a sequence similarity to yeast Prp8 protein. This gene is a candidate gene for autosomal dominant retinitis pigmentosa (provided by RefSeq, July 2008).
GI_37540877-S	GLCE	Glucuronic acid epimerase	15q23	HSEPI	Enables calcium ion binding activity; heparosan-N-sulfate-glucuronate 5-epimerase activity; and protein homodimerization activity. Involved in heparan sulfate proteoglycan biosynthetic process. Predicted to be located in Golgi membrane. Predicted to be integral component of membrane. Predicted to be active in Golgi apparatus (provided by Alliance of Genome Resources, April 2022).
GI_28872783-A	CDK5RAP1	CDK5 regulatory subunit associated protein 1	20q11.21	C42; CGI-05; HSPC167; C20orf34	This gene encodes a regulator of cyclin-dependent kinase 5 activity. This protein has also been reported to modify RNA by adding a methylthio-group and may thus have a dual function as an RNA methylthiotransferase and as an inhibitor of cyclin-dependent kinase 5 activity. Alternative splicing results in multiple transcript variants that encode different isoforms (provided by RefSeq, May 2013).
GI_14589948-S	POLR2A	Polymerase (RNA) II (DNA directed) polypeptide A, 220 kDa	17p13.1	RPB1; RPO2; POLR2; POLRA; RPBh1; RPOL2; NEDHIB; RpIILS; hsRPB1; hRPB220	This gene encodes the largest subunit of RNA polymerase II, the polymerase responsible for synthesizing messenger RNA in eukaryotes. The product of this gene contains a carboxy terminal domain composed of heptapeptide repeats that are essential for polymerase activity. These repeats contain serine and threonine residues that are phosphorylated in actively transcribing RNA polymerase. In addition, this subunit, in combination with several other polymerase subunits, forms the DNA binding domain of the polymerase, a groove in which the DNA template is transcribed into RNA (provided by RefSeq, July 2008).
GI_23503234-A	C1QDC1	caprin family member 2	12p11	EEG1; EEG-1; C1QDC1; RNG140	The protein encoded by this gene may regulate the transport of mRNA. It may play a role in the differentiation of erythroblasts. Multiple transcript variants encoding different isoforms have been found for this gene (provided by RefSeq, February 2016).

## 4 Discussion

The simulation experiments provide a comprehensive evaluation across the three methods with 1,000 repetitions of large-scale trials comprised of 1,000 cases and 1,000 controls each and nearly ‘ideal’ subtype biomarkers representing each subtype percentage. The results from these trials are stunning.

In general, FC performed extremely poorly. Even when 50% of the cases were associated with the subtype biomarker, the median log2FC value was only 0.608. The maximum across all 1,000 trials was 0.821. Consequently, all of the pseudo biomarkers would be discarded, despite their nearly perfect discrimination of a subtype comprised of half of the cases. Moreover, every one of the biomarkers would be discarded for all the other scenarios.

It’s trickier to evaluate AUC, due to lack of a clear significance cutoff value. The literature points to 0.7 or 0.75 and our trials on AD gene expression provided a cutoff of 0.74 for *p*-value ≤ 0.05. All the pseudo biomarkers had AUC values less than 0.70 across the 1,000 trials for subsets less than 40%. Furthermore, the median for the 40% subset trial was 0.695. Significance emerged as the subset size grew to 50%.

Being a newly introduced metric, there is no established significance cutoff for BCD. The AD gene expression data provided a cutoff value of 0.209 for p-value ≤0.05. Using this proxy, *every* one of the 1,000 trials for subsets of 10% or more would be marked as significant. Even a few of the trials with subset size of 5% were above 0.209. These results document the power of the use of the distribution, rather than the magnitude, of data values to identify subtypes within a population.

As expected for the biological trials, the top six genes identified by FC and AUC show significant differences between the diseased cases and normal controls (Figures 4, 5). While several of the top results exhibit some degree of bimodality, others tend towards differences across the majority of the samples. On the other hand, each top BCD result clearly delimitates a subgroup, without requiring aberrant levels for individuals that are not in the given subgroup (Figure 6).

A particularly interesting result is that the first round of BCD trials produced spurious associations for the top six values due to the imbalance of cases and controls samples from brain region 4. While this imbalance, coupled with differential expression across brain regions, created clear subsets, none of these genes were included in the top six genes for FC or AUC in the first round of trials.

The six most significant genes identified by BCD include four genes previously associated with AD and two novel genes. Genes that have known associations with AD include *CHGB* (Lechner et al., 2004; Quinn et al., 2020; Marksteiner et al., 2002; Willis et al., 2008), *GLCE* (Perez-Lopez et al., 2021; Ozsan McMillan et al., 2023; Liachko et al., 2019; Schultheis et al., 2021), *CDK5RAP1* (Esteras et al., 2012) and the gene it regulates, *CDK5* (Liu et al., 2016; Maccioni et al., 2001; Shukla et al., 2012; Tsai et al., 2004; Cruz and Tsai, 2004; Monaco III, 2005; Maitra and Vincent, 2022; Nikhil et al., 2019; Lau and Ahlijanian, 2003; Pei et al., 1998), and *POLR2A* (Dickson et al., 2021). Chromogranin B (CHGB) has been observed in about 60% of the amyloid-beta plaques in AD transgenic mice and these mice performed poorly in the Morris water maze task (Willis et al., 2008). This protein has been proposed as a synaptic degeneration marker for AD (Marksteiner et al., 2002). Glucuronic acid epimerase (GLCE) modifies heparan sulfate by converting the glucuronic acid to iduronic acid. This gene is downregulated in AD (Sepulveda-Diaz et al., 2015; Huynh et al., 2019) and suggested to contribute to the aberrant behavior of heparan sulfate in AD (Perez-Lopez et al., 2021; Ozsan McMillan et al., 2023). CDK5 regulatory subunit-associated protein 1 (CDK5RAP1) is involved in checkpoint and arrest in the cell cycle as it inhibits CDK5, a protein with strong implications for AD progression (Liu et al., 2016; Maccioni et al., 2001; Shukla et al., 2012; Tsai et al., 2004; Cruz and Tsai, 2004; Monaco III, 2005; Maitra and Vincent, 2022; Nikhil et al., 2019; Lau and Ahlijanian, 2003; Pei et al., 1998). Esteras et al. observed significant upregulation of CDK5RAP1 in AD transgenic mouse brain (1.98 fold change) and PBMCs (10.69 fold change) (Esteras et al., 2012). The largest subunit of RNA polymerase II, POLR2A, also known as RPB1, has recently been linked to AD by Dickson et al. Using an AD transgenic mouse model, this group demonstrated the mislocalization of this protein from the nucleus to the cytoplasm in a tau-and age-dependent manner (Dickson et al., 2021).

*PRPF8* currently has no obvious connection to AD, however, it was one of 10 genes found to be associated with AD and Parkinson’s disease in another study involving a different dataset (GSE4229) using an alternative tissue: peripheral blood (Faruqui et al., 2021). Finally, *C1QDC1* is another novel gene without any clear association. Table 3 includes descriptions of the six genes.

FC identified one of the six BCD-significant genes, *CHGB*, with a *p*-value of 0.0079. The other five were missed by FC and all six were missed by AUC. In general, BCD is able to tease out novel genes missed by the other methods as FC and AUC shared 46.1% of their significant genes while only 3.7 and 4.6% of the BCD genes were identified by FC and AUC, respectively (Supplementary Tables S2–S4).

It should be noted that BCD is not expected to identify global biomarkers. When nearly all of the cases are associated with the biomarker, e.g., pTau-181 associations with AD, a shift in the cases median and mean, not modality, is expected. Such biomarkers are better captured using FC or AUC as medians and means are ignored by BCD.

It should also be noted that the lower bound on sample size for BCD is limited by the ability to distinguish the Bimodality Coefficient for the distributions. Since this statistic is derived from the skewness and kurtosis of the data, an adequate sample size for each of these statistics is requisite. Furthermore, the Bimodality Coefficient includes the sample size in its formulation.

BCD enjoys the same favorable properties exhibited by AUC. No specific biomarker threshold or other parameters are utilized. The metric is simple and intuitive. Furthermore, examination of the corresponding histograms provides additional information beyond the scalar value. As a bonus, individuals representing the subtype are distinguished from those who are not associated.

At the same time, BCD does not suffer from AUC’s drawbacks. AUC includes regions under the curve where analyte thresholds are not of practical interest and can be misleading when comparing two ROC curves that cross. Neither of these issues are of concern for BCD as the distributions of analyte levels, rather than TPRs and FPRs, dictate the computed values. Furthermore, high AUC value does not always correlate with the ability to identify a robust threshold for practical use of the biomarker. In contrast, high BCD value indicates strong bimodality, which corresponds to a natural inversion between the modes. The horizontal axis values delineate the corresponding threshold. Finally, analytes that are already known to be unimodal under normal conditions do not necessarily require any new controls data to be generated and ranks of the BC values for the cases across the whole set of tested analytes can be used to distinguish significance.

AD is a complex and heterogeneous disease and identification of subtypes is needed to advance precise treatments of each subtype group. We demonstrate here that popular statistics used for assessing biomarkers, FC and AUC, generally perform suboptimally when heterogeneity exists. We also provide a new metric, BCD, which appears to hold promise in this domain.

## Data Availability

Publicly available datasets were analyzed in this study. This data can be found here: https://www.ncbi.nlm.nih.gov/geo/query/acc.cgi?acc=GSE15222.
