# Supplementary material for: Capturing biomarkers associated with Alzheimer disease subtypes using data distribution characteristics
Source: Front Comput Neurosci. 2024 Sep 3;18:1388504. doi: 10.3389/fncom.2024.1388504 (PMC11413970; doi:10.3389/fncom.2024.1388504)
Supplement: Supplementary file 6 [file Data_Sheet_1.pdf]

frontiers

Supplementary Material

1 Determinations of means and standard deviations for simulated data

The mean and standard deviations utilized for the two distributions in our simulated trials were derived from analyzing a proteomics data set that we previously generated(Wang *et al.*, 2023). A cohort of 192 COVID-19 positive patients was separated into severe and non-severe groups based on hospitalization status, resulting in 92 severe and 100 non-severe individuals. Individuals that died from COVID-19 or were in the ICU due to COVID-19 were considered severe. Using the median values from these groups, the fold change (FC) of each protein was calculated, where the non-severe group was treated as the reference group. Additionally, a p-value associated with each FC was derived using the Mann-Whitney U statistic.

The original set of 4634 proteins was reduced to 383 highly differentially-expressed proteins, by removing those with p-values < 1e-6 or $|\log_2(FC)| < 3$. These proteins were scaled using min-max normalization, and the resulting median and standard deviations were calculated for each of the two groups. The average of these four values were used as parameters in the simulated normal distributions: μ_1=0.03, σ_1=0.04, μ_2=0.40, σ_2=0.16.

References

Wang, L. *et al.* (2023) 'Plasma proteomics of SARS-CoV-2 infection and severity reveals impact on Alzheimer's and coronary disease pathways', *iScience*. Elsevier Inc., 26(4). doi: 10.1016/j.isci.2023.106408.
